# Current state of dental informatics in the field of health information systems: a scoping review

**DOI:** 10.1186/s12903-022-02163-9

**Published:** 2022-04-19

**Authors:** Ballester Benoit, Bukiet Frédéric, Dufour Jean-Charles

**Affiliations:** 1 Pôle d’Odontologie, Assistance Publique des Hôpitaux de Marseille, Marseille, France; 2 Aix Marseille Univ, CNRS, ISM, Inst Movement Sci, Marseille, France; 3 Aix Marseille Univ, INSERM, IRD, SESSTIM, Sciences Economiques & Sociales de la Santé & Traitement de l’Information Médicale, ISSPAM, Marseille, France; 4 APHM, Hôpital de la Timone, Service Biostatistique et Technologies de l’Information et de la Communication, Marseille, France

**Keywords:** Dental informatics, Health information systems, Electronic health records, Clinical coding, User-computer interface, Data capture, Medical data reuse

## Abstract

**Background:**

Over the past 50 years, dental informatics has developed significantly in the field of health information systems. Accordingly, several studies have been conducted on standardized clinical coding systems, data capture, and clinical data reuse in dentistry.

**Methods:**

Based on the definition of health information systems, the literature search was divided into three specific sub-searches: “standardized clinical coding systems,” “data capture,” and “reuse of routine patient care data.” PubMed and Web of Science were searched for peer-reviewed articles. The review was conducted following the PRISMA-ScR protocol.

**Results:**

A total of 44 articles were identified for inclusion in the review. Of these, 15 were related to “standardized clinical coding systems,” 15 to “data capture,” and 14 to “reuse of routine patient care data.” Articles related to standardized clinical coding systems focused on the design and/or development of proposed systems, on their evaluation and validation, on their adoption in academic settings, and on user perception. Articles related to data capture addressed the issue of data completeness, evaluated user interfaces and workflow integration, and proposed technical solutions. Finally, articles related to reuse of routine patient care data focused on clinical decision support systems centered on patient care, institutional or population-based health monitoring support systems, and clinical research.

**Conclusions:**

While the development of health information systems, and especially standardized clinical coding systems, has led to significant progress in research and quality measures, most reviewed articles were published in the US. Clinical decision support systems that reuse EDR data have been little studied. Likewise, few studies have examined the working environment of dental practitioners or the pedagogical value of using health information systems in dentistry.

**Supplementary Information:**

The online version contains supplementary material available at 10.1186/s12903-022-02163-9.

## Background

Advances in dentistry largely depend on developments in information technology. Introduced by Zimmerman *et al.* in 1968 [1], “dental informatics” refers to the application of computer and information sciences to dentistry with the aim of improving clinical practice, research, education, and management [2, 3].

Some of the advances made in dental informatics include applications such as diagnostic devices, 2D or 3D digital acquisition, computer-assisted design and manufacturing, and computer-assisted surgery [4, 5]. These advances were examined in a systematic review published in 2017 [6].

Other studies have investigated the development of computerized health information systems (HISs) in dentistry. Health information systems are used to collect, store, process, and transmit the information needed to organize and implement care [4, 7]. One well-known HIS component is the electronic dental record (EDR), which is used by practitioners to document both patients’ medical and dental history and detailed information on consultations. Given the obvious benefits of EDRs, especially in the context of large clinical institutions, EDR overall adoption rate in the US increased from 52% in 2012 [8] to 77% in 2017 [9]. Indeed, EDRs are not a simple transposition of paper records. Ideally interoperable with other HIS components, they allow to control data capture, facilitate data storage and access, support administrative and management processes, and guide public health policies. They can also be used in research and education [10–13].

To take full advantage of EDRs, particularly with regards to the communication, aggregation, and reuse of data, standardized clinical coding systems (SCCSs) are needed that are scalable, shareable, and adapted to the dentistry domain [13]. Such systems make it easier machine-readable documentation, and allow for computerized comparisons of the outcomes of different treatments for the same diagnosis [13, 14].

In the early 2000s, no consensus-standardized nomenclature for dental diagnoses and treatment outcomes was available [13]. The lack of a collective strategy among governments, health centers, and software developers strongly limited progress in this area [15]. Moreover, the most widely used coding system at the time, the International Classification of Diseases (ICD-9 [16]), provides limited coding for dentistry and is inadequate for making appropriate dental diagnoses [17]. Several studies have attempted to overcome this problem by proposing different coding systems, including EZCode [18], SNODENT, Ontology for Dental Research [19], Oral Health and Disease Ontology [20], etc. The available literature on coding systems in dentistry is confusing at first glance. Some of the proposed classifications have been redefined or renamed several times, and, in some cases, they have been merged with other coding systems to fit the needs of dentistry.

In addition to standardization issues, clinical dental coding usage is mainly considered as an academic concern whereas private practitioners should also be concerned by this [2]. Difficulties in capturing standardized data may explain why private practitioners in dentistry have failed to adopt SCCSs. Indeed, making the capture of standardized data more efficient is essential to improve usability, workflow integration, but also data quality.

The reuse of EDR data holds much promise in the area of research. Not only can it diminish the costs and inefficiencies associated with clinical research, but shared EDR data warehouses can surpass many registries and data repositories in volume. Moreover, EDRs are real-world data sources that can be used in studies to produce real-world evidence, which in turn can help accelerate advances in care, improve outcomes for patients, and provide important insights for daily practice. Like other forms of retrospective research, EDR-based retrospective studies require neither patient recruitment nor the collection of new data, both of which are expensive and time-consuming. While the reuse of EDR data is a promising step towards decreasing research costs, facilitating patient-centered research, and speeding the rate of new medical discoveries [21], it is nevertheless limited by data quality concerns. Indeed, it is generally accepted that due to differences in priorities between clinical practice and research, clinical data are not recorded with the same care as research data [21]. Thus, in addition to proper ergonomics and workflow integration, there is a need for data capture forms that can intercept basic errors and provide real-time feedback to keep the user informed of what is going on, thus ensuring that the most accurate and complete information is collected.

In addition, EDR data can be used in clinical decision support systems (CDSSs) to provide real-time patient-centered clinical recommendations. They can also be used in educational settings to monitor students’ technical and theoretical knowledge as well as their clinical activity.

The primary objective of this scoping review was to summarize studies on SCCSs and EDR data capture in dentistry. The secondary objective was to explore the practical implications of reusing EDR data in CDSSs, quality measure development and clinical research.

## Methods

Based on the definition of HISs [22], the literature search was divided into three specifics searches:

1. *HISs in dentistry* and SCCSs
2. *HISs in dentistry* and data capture
3. *HISs in dentistry* and reuse of routine patient care data

For each of the searches, a review of the literature was conducted in August 2020 and then updated on January 2021 following the Preferred Reporting Items for Systematic Reviews and Meta-Analyses extension for Scoping Reviews (PRISMA-ScR) protocol. PubMed was used to search the MEDLINE bibliographical database, and Web of Science was used to search all databases. Studies published between 1 January 2000 and 9 January 2021 were selected. This long period of inclusion helped to account for developments in the field and for the current state of knowledge on each of the subjects discussed.

The search strategies used in this scoping review are presented in Table 1. Studies were selected according to the inclusion criteria established for each search (Table 1).

**Table 1:** Search strategies

	Health information systems in dentistry and standardized clinical coding systems	Health information systems in dentistry and data capture	Health information systems in dentistry and reuse of routine patient care data
Database	PubMed (MEDLINE) and Web of Science (all databases)		
Publication date	1 January 2000–present (9 January 2021)		
Keywords	Diagnostic terminology/codes/system, standardized terminology, clinical coding	Data capture, user-computer interface	Quality Measurement, Clinical Decision Support Systems, Data Warehouse
Inclusion criteria	Scientific articles dealing with standardized clinical coding systems	Scientific articles dealing with data capture	Scientific articles dealing with reuse of routine patient care data
Exclusion criteria	Publication not in English language; study not specifically related to health information systems in clinical dentistry; study using electronic dental record data without consideration of health information systems; publication in the form of a letter, editorial/opinion, abstract, conference abstract, case report, or book chapter		
PubMed Final search	("dental diagnostic system"[TW] OR "diagnostic terminology"[TW] OR "diagnostic codes"[TW] OR "standardized terminology"[TW] OR "Clinical Coding"[Mesh]) AND ("Dental Informatics"[Mesh] OR "Dental Informatics"[TW] OR "dentistry"[TIAB] OR "dental"[TIAB]) AND ("2000/01/01"[Date - Publication]: "3000"[Date - Publication])	("data capture" OR (("Surveys and Questionnaires"[MeSH] OR "forms and records control"[MeSH] OR "Patient Health Questionnaire"[MeSH] OR "Form"[MeSH] OR "Records"[MeSH] OR "user-computer interface"[MeSH]) AND "Electronic Health Records"[MeSH])) AND ("Dental Informatics"[Mesh] OR "Dental Informatics"[TW] OR "dentistry"[TIAB] OR "dental"[TIAB]) AND ("2000/01/01"[Date - Publication]: "3000"[Date - Publication])	("reusing electronic patient data" OR "Quality Measurement" OR "data repository" OR " Clinical Decision Support Systems" OR "data warehouse") AND ("Dental Informatics"[Mesh] OR "Dental Informatics"[TW] OR "dentistry"[TIAB] OR "dental"[TIAB]) AND ("2000/01/01"[Date - Publication]: "3000"[Date - Publication])
Web of Science Final search	TS = (("dental diagnostic system" OR "standardized diagnostic terms" OR "dental coding" OR "diagnostic terminology" OR "Diagnostic Codes" OR "standardized terminology" OR "Clinical Coding") AND ("dentistry" OR "dental")) Databases = WOS, CCC, KJD, MEDLINE, RSCI, SCIELO Timespan = 2000–2021	TS = (("data capture" OR "Surveys and Questionnaires" OR "forms and records control" OR "Patient Health Questionnaire" OR "Form" OR "Records" OR "user-computer interface") AND ("dentistry" OR "dental") AND ("Electronic Health Records")) Databases = WOS, CCC, KJD, MEDLINE, RSCI, SCIELO Timespan = 2000–2021	TS = (("reusing electronic patient data" OR "Quality Measurement" OR "data repository" OR " Clinical Decision Support Systems" OR "data warehouse") AND ("Dental Informatics" OR "Dental" OR "dentistry")) Databases = WOS, CCC, KJD, MEDLINE, RSCI, SCIELO Timespan = 2000–2021
**Articles found**	Pubmed: 67 Web of Science: 146	Pubmed: 224 Web of Science: 375	Pubmed: 81 Web of Science: 98

Studies were excluded if they met any of the following criteria: (a) publication not in English; (b) study not specifically related to health information systems in clinical dentistry; (c) study using only EDR data without consideration of HISs; (d) publication in the form of a letter, editorial/opinion, abstract, conference abstract, case report, or book chapter.

For each search, duplicates were removed, and articles were initially selected based on their titles and abstracts. When the abstract did not provide sufficient information, the full text was read. All stages of the search were carried out by the authors and then carefully checked to minimize bias in the review process. In case of disagreement, the decision was made by consensus.

**Fig. 1:**
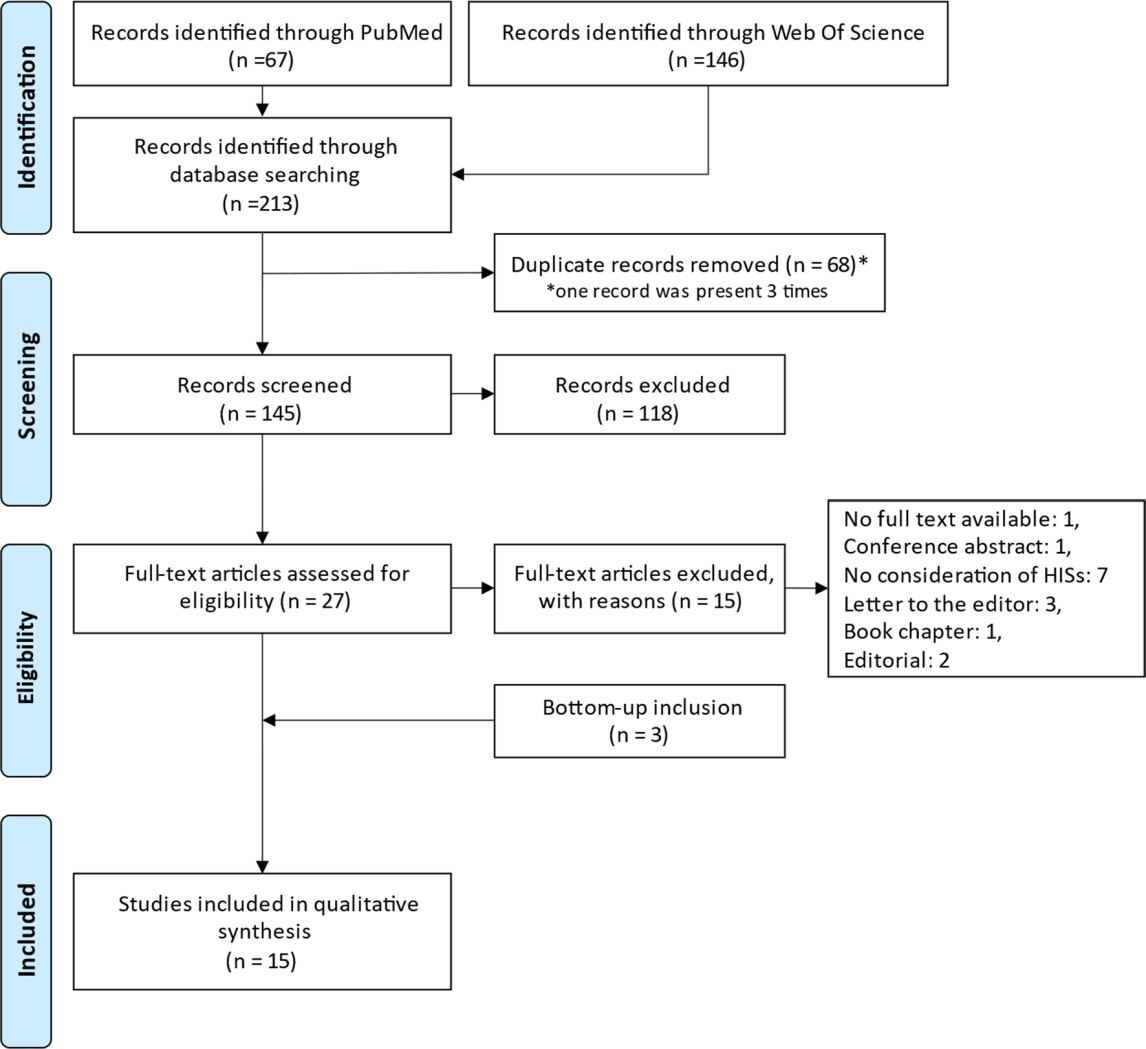
PRISMA flow diagram of the search for studies on health information systems in dentistry and standardized clinical coding systems

**Fig. 2:**
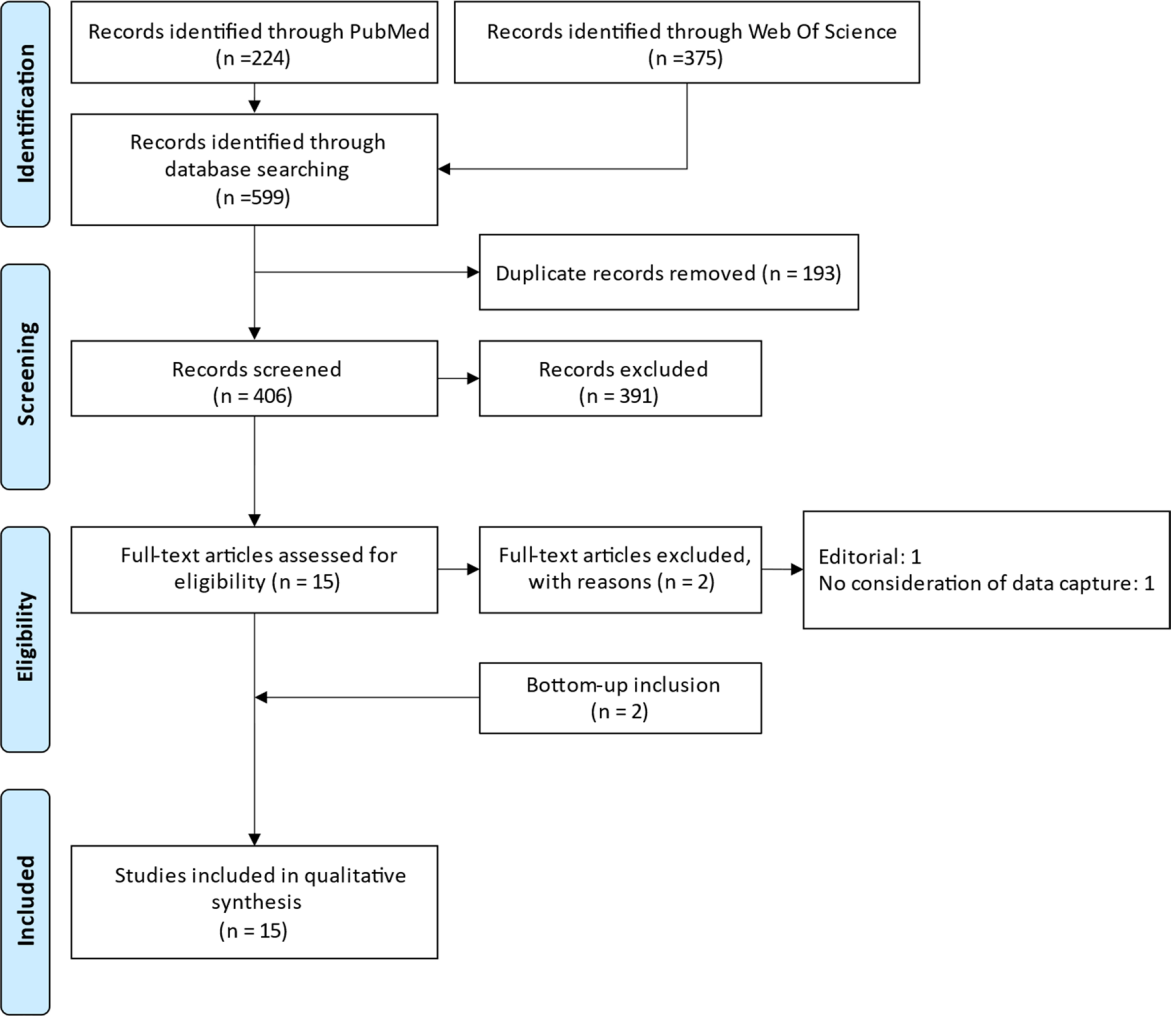
PRISMA flow diagram of the search for studies on health information systems in dentistry and data capture

**Fig. 3:**
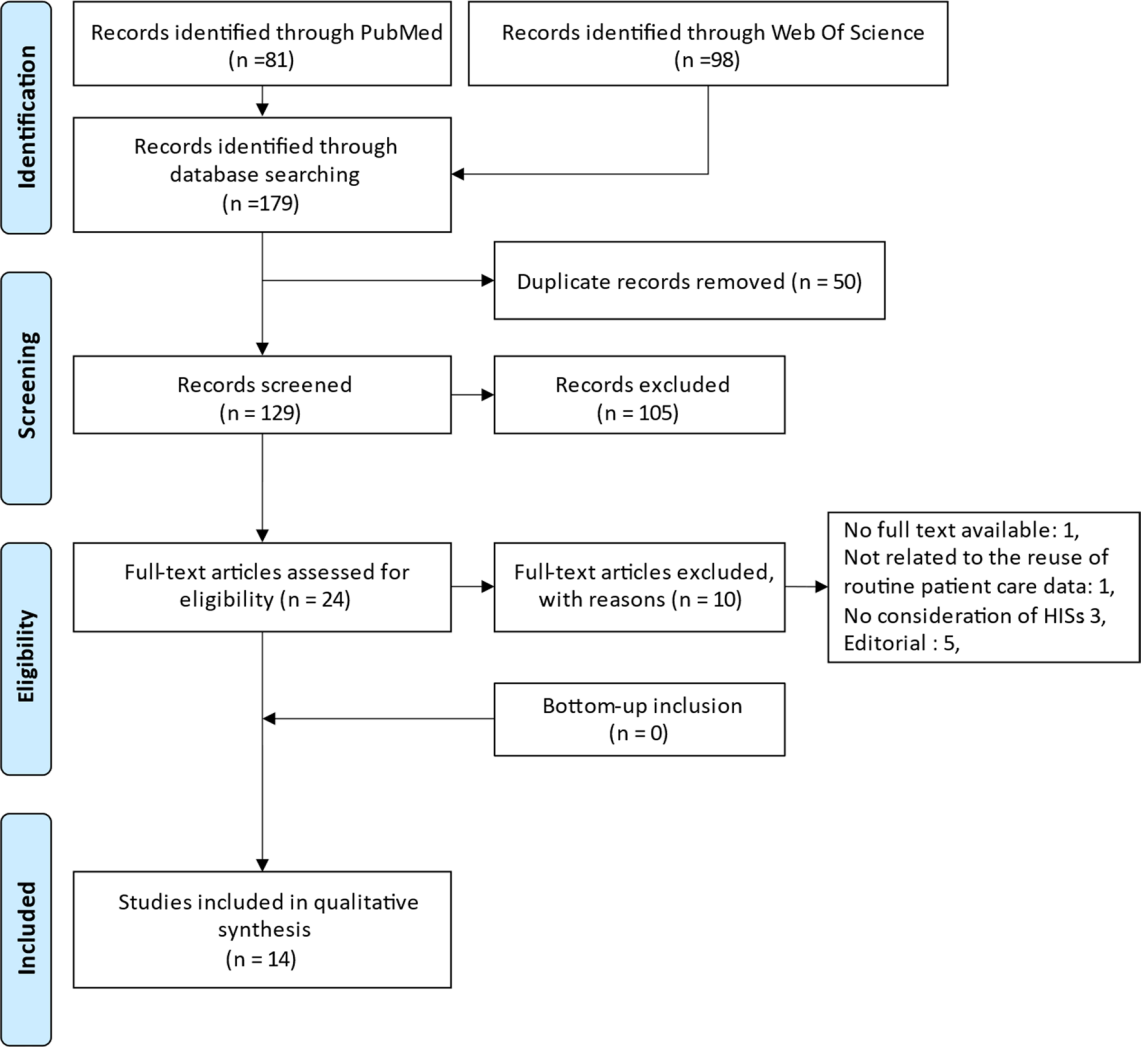
PRISMA flow diagram of the search for studies on health information systems in dentistry and reuse of routine patient care data

The flow diagrams of the three searches are summarized in Figs. 1, 2, and 3.

Only the first affiliation of the first author was considered in the geographical analysis of publications.

## Results

A total of 44 articles were selected for review. As regards geographical distribution, 31 articles (70%) had a first author affiliated with the United States, and the other 13 articles (30%) had a first author affiliated with Australia, Canada, China, Czech Republic, India, the Netherlands, Poland, Saudi Arabia, Sweden, or the United Kingdom.

### Health information systems in dentistry and standardized clinical coding systems

Table 2 summarizes the articles selected in the first search.

**Table 2:** Articles selected in the search on “health information systems in dentistry and standardized clinical coding systems”

References	SCCS-related themes	Purpose(s)
Leake 2002 [23]	General	Overview of the definition, utility, and developments to date of diagnostic codes in dentistry
Goldberg 2005 [24]	Internal quality evaluation	Computer-based evaluation of SNODENT’s internal quality
Smith et al*.,* 2010 [19]	Design/development	Introduction of an ontology based on the OBO Foundry
White et al*.*, 2011 [18]	Usage evaluation & validation	EDR-based evaluation of Z code usage in predoctoral clinical practice in a US dental school
Kalenderian et al*.*, 2011 [17]	Design/Development	Development of EZcodes [later renamed DDS] through iterative process by a work group of dental faculty members
Tokede et al*.*, 2013 [25]	Usage evaluation & validation	EDR-based evaluation of EZcodes [DDS] usage over a 1-year period in 3 dental schools
Schleyer et al*.*, 2013 [20]	Design/Development	Introduction of the Oral Health and Disease Ontology based on the OBO Foundry
Lam et al*.*, 2014 [2]	Design/Development	Extension of billing codes to include diagnostic information
Reed et al*.*, 2015 [26]	Pedagogical evaluation	Case–control multicentric study in 3 dental schools to determine whether exposure to DDS terms improves students’ scores in the Health Sciences Reasoning Test
Ramoni et al*.*, 2015 [27]	User perception	Electronic survey of attitudes and beliefs toward the use of dental diagnostic terminology in the US
Ramoni et al*.*, 2017 [14]	Usage description	Electronic survey of standardized dental diagnostic terminology usage in US dental schools
Sutton et al*.*, 2017 [28]	Usage evaluation & validation	Case-based evaluation of DDS usage for radiographic carious lesions among dental faculty members
Obadan-Udoh et al*.*, 2017 [29]	User perception	Survey of users’ attitudes towards standardized dental diagnostic terminologies and evaluation of strategies to improve their use
Yansane et al*.*, 2019 [30]	Usage evaluation & validation	EDR-based evaluation of DDS usage over a 4-year period in 5 dental institutions
Taylor et al*.*, 2019 [31]	Usage evaluation & validation	Case-based evaluation of SNODENT usage among dental faculty members and students

SCCS: Standardized Clinical Coding System; OBO: Open Biomedical Ontologies; EDR: Electronic Dental Record; DDS: Dental Diagnostic System

#### Design and development of standardized clinical coding systems

**Fig. 4:**
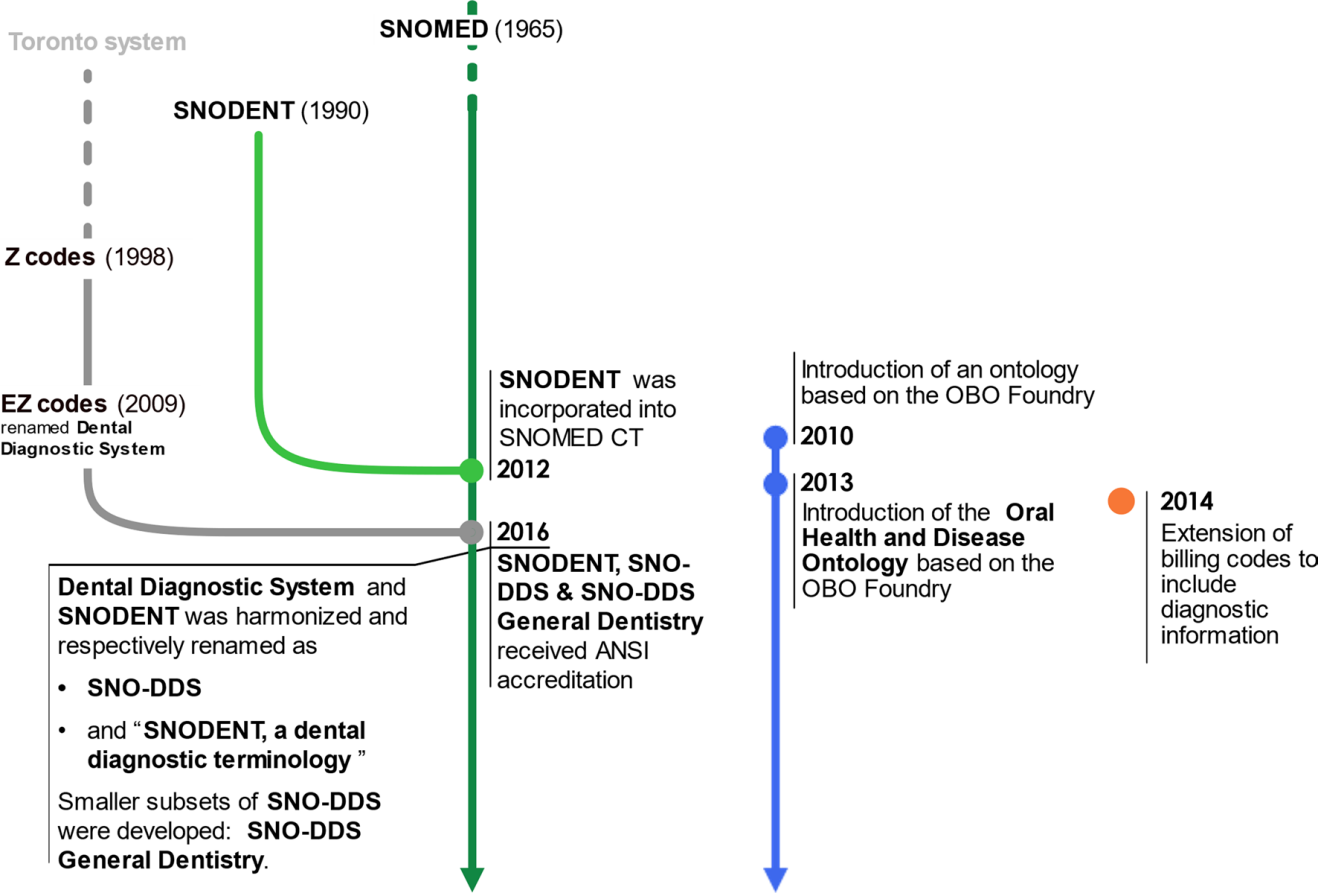
Design and development of standardized clinical coding systems [2, 14, 17–20, 23, 25, 32]. DDS: dental diagnostic system; OBO: open biomedical ontologies; ANSI: American National Standards Institute

Articles [2, 14, 17–20, 23, 25, 32] trace the design and development of SCCSs in dentistry, as shown in Fig. 4.

All SCCSs examined in this scoping review are independent of national procedures codification systems except for that proposed by Lam et al. [2]. As a result of evolution over time, only two SCCSs are available nowadays:

- SNOMED (and its subsets)—license-based access.
- The Oral Health and Disease Ontology designed by Schleyer et al. [20] on the Open Biomedical Ontologies (OBO) Foundry framework [33]—freely available.

#### Evaluation and validation of standardized clinical coding systems

In 2005, Goldberg et al*.* [24] used a software-based method to evaluate the internal quality of SNODENT. This method consisted of searching for errors in ontology by comparing different ways of extracting information from terms, concepts, descriptions, and definitions. The authors found that SNODENT had quality issues, mainly due to confusion between terms and concept codes (for example, unclear relationships between terms and concepts, polysemic concepts, subsumption problems, etc.).

Practice-based evaluations were performed only for Z codes [18], the Dental Diagnostic System (DDS) [25, 28, 30], and SNODENT [31]. Some studies evaluated diagnostic code entry by determining the plausibility of the entered diagnostic code based on the entered treatment procedure code [18, 25, 28, 30], while others compared written diagnoses to diagnostic codification [31].

The validation scores obtained in these studies are provided in Table 3.

**Table 3:** Evaluation and validation of standardized clinical coding systems

References	Coding system	Study method	Mandatory usage	Usage	Correct coding (%)
White et al*.*, 2011 [18]	Z codes	EDR-based evaluation in a predoctoral clinic over a 1-year period	No	38.9%	76.7%
Tokede et al*.*, 2013 [25]	DDS	EDR-based multicentric evaluation in predoctoral clinics over a 1-year period	No	12%	60.5%
Sutton et al*.*, 2017 [28]	DDS	Case-based evaluation among general dentistry faculty members	NA	NA	Between 54.7% and 89.3% depending on clinical case
Yansane et al*.*, 2019 [30]	DDS	EDR-based multicentric evaluation in 4 academic research centers and 1 private clinic over a 4-year period	Yes	between 80 and 100%	From 77.7% to 86.2% over the study period
Taylor et al*.*, 2019 [31]	SNODENT	Case-based evaluation among students and faculty members	NA	NA	64.5%

EDR: Electronic Dental Record; DDS: Dental Diagnostic System

The selected studies highlighted different kinds of errors and explored various avenues for the improvement of SCCS usage.

Some studies found that the more the SCCS is comprehensive, the less it is easy to navigate and the more complicated it is for the practitioner to use [18, 25]. In addition, videos analyses have shown that practitioners find it sometimes difficult to determine which code or concept represents the right choice, especially when different codes or concepts have similar meanings [31].

The modes by which diagnostic terms are found and entered were also shown to be a source of error. Accordingly, a human–machine interface that can support accurate and complete SCCS-based documentation should be developed. Moreover, data capture forms should be made more intuitive, quick, and easy to use. Lastly, control mechanisms with appropriate user feedback should be put in place to limit common errors [25, 31].

Finally, errors may be due to practitioners themselves, as these often have insufficient knowledge of SCCSs. Tokede et al*.* [25] highlighted practitioners’ lack of awareness of the impact of using standardized vocabulary. On the other hand, Yansane et al*.* [30] showed that practitioners are becoming increasingly familiar with EDRs and SCCSs as they get to use them, and that this improves user experience and the quality of data entry. Coding errors may also reflect the miscalibration of diagnostic criteria. In their study based on clinical cases of carious lesions, Sutton et al*.* [28] found that participants were just as likely to choose an incorrect diagnostic code as the correct one when recording cases of enamel-limited lesions. In their view, this suggests both the need for faculty calibration (particularly in the field of diagnostics) and the educational value of using diagnostic codes.

In 2015, Reed et al*.* [26] evaluated the impact of SCCS exposure on dental faculty students’ scores in the Health Sciences Reasoning Test (HSRT). The HSRT is designed to measure critical thinking skills, and is specifically calibrated for health science practitioners and students in health science educational programs. The authors showed that students exposed to SCCSs (in this case the DDS) had a significantly higher Health Sciences Reasoning Test score than those who had not.

#### Adoption of standardized clinical coding systems in academic settings

In 2017, Ramoni et al. [14] conducted a survey of US dental school deans about their usage of SCCSs. The response rate was 57% (35/61). A total of 32 deans reported using an EDR to document patient care and for administrative purposes such as billing; of these, 84% used the AxiUm EDR system. Twenty-nine deans were familiar with the DDS, but only ten had loaded it into their EDR for clinical use. Two schools used a self-selected subset of the SNODENT ontology, and five schools used the dental terminology of the ICD, 9th Revision [16].

To the authors’ knowledge, only one large private clinic had implemented the DDS in the US in 2017. By contrast, the DDS was recommended as standard SCCS in the Netherlands in 2015 [14].

#### User perception of standardized clinical coding systems

Two articles assessed users’ perceptions of SCCS (see Table 4) [27, 29]. They both concluded that users have a positive attitude towards standardized diagnostic terminologies (DxTMs). In another survey by Ramoni et al*.* [27], the highest average score on the Likert scale (a psychometric tool used to measure the degree of agreement with a proposal) was associated with the item “Standardized dental diagnostic terms would allow dental team members to use the same term to describe the same diagnosis” followed by “standardized dental diagnostic terms would be useful.” However, 16% of the responses reflected confusion about what standardized dental diagnostic terminology entails, and participants expressed doubt that the use of SCCSs would result in better dental care [27].

**Table 4:** User perception of standardized clinical coding systems

References	Type of study	Perception measurement	Respondents/participants
Ramoni et al*.*, 2015 [27]	Email survey of US private dental group	Assessment of participants’ attitudes towards standardized dental diagnostic terms using a questionnaire with a 5-point Likert scale and an open-ended question	749 respondents (68% of whom were clinical staff and 32% were not)
Obadan-Udoh et al*.*, 2017 [29]	US conference breakout session	Assessment of participants’ attitudes towards diagnostic terminology usage and proposal of strategies to improve SCCS usage	82 academic and non-academic participants

SCCS: Standardized Clinical Coding System

In the study by Obadan-Udoh et al*.* [29], academic and non-academic users proposed a series of strategies to improve SCCS usage. These strategies can be divided into three types:

- Political strategies, including usage obligation and financial incentives.
- Educational strategies, including user training on the purpose and benefits of SCCSs.
- Technical strategies, including smooth incorporation of SCCSs into EDRs with easier user interface and streamlined workflow.

### Health information systems in dentistry and data capture

Table 5 presents the articles selected in the second search.

**Table 5:** Articles selected in the search “health information systems in dentistry and data capture”

References	Data capture-related themes	Purpose(s)
Chadwick et al*.*, 2002 [34]	Capture technique	Assessment of the use of barcodes to record clinical activity in dental schools
Thyvalikakath et al*.*, 2008 [35]	Interface usability	Assessment of interface usability in 4 commercial dental computer-based patient record systems
Irwin et al*.*, 2009 [36]	Capture technique	Development and evaluation of a semantic representation for natural language processing
Hippmann et al*.*, 2010 [37]	Capture technique	Introduction of a voice supported EDR in the field of temporomandibular joint disorders
Hill et al*.*, 2010 [38]	General, workflow integration	Assessment of the impact of integrating health information technology systems into chair-side patient care on dental school users
Walji et al*.*, 2013 [39]	Interface usability	Detection and characterization of usability problems in structured data entry interfaces in dentistry
Tancredi et al*.*, 2013 [40]	Interface usability	Application of the semiotic inspection method to assess the interface usability of an EDR
Noureldin et al*.*, 2014 [41]	Data completeness	Quality assessment of care data documentation in an EDR in primary health care units of Alexandria, Egypt
Walji et al*.*, 2014 [42]	Interface usability	Evaluation of the effectiveness of 3 different methods for the detection of usability problems in an EDR: user testing, semi-structured interviews, and surveys
Thyvalikakath et al*.*, 2014 [43]	Interface usability, Workflow integration	Assessment of dentist workflow during a typical patient examination to help design a novel EDR interface
Tokede et al*.*, 2016 [10]	Data completeness	Assessment of data entered in an EDR and of the frequency of update of each clinical entry (Delphi process)
Schwei et al*.*, 2016 [44]	Workflow integration	Assessment of EDR workflow using time and motion methodology to identify breakdowns and opportunities for process improvement
Thierer et al*.*, 2017 [45]	Data completeness	Assessment of the improvement of progress note documentation by dental students after an educational intervention
Sidek et al*.*, 2017 [11]	General	Identification of the perceived critical success factors of EDR system implementation in a dental clinic
Mishra et al*.*, 2019 [46]	Capture technique	Development of a natural language processing application to automatically annotate clinical notes with unified medical language system codes

EDR: Electronic Dental Record

#### Data completeness

Good record keeping is a fundamental professional and legal obligation. However, studies conducted in Australia, the United Kingdom, Scandinavian countries, and Egypt show that clinical dental record keeping practices do not meet basic standards [10, 41]. Thus, a 2000 study revealed that patient clinical information was absent in 9.4% to 87.1% of EDRs [45]. In 2016, Tokede et al. [10] used a Delphi process to determine what data should be entered in dental records and how often each clinical entry should be updated (Additional file 1: Appendix A). In so doing, they emphasized the need for consensus on what data is necessary. The assumption is that practitioners are less likely to record information deemed unimportant or worthless, causing the problem of incomplete or inaccurate data entry to persist [10]. However, not taking into consideration practitioners’ information needs can lead to organizational difficulties, prompting the use of both paper and electronic forms for data documentation. In this regard, it should be noted that Thierer et al*.* found that the rate of documentation of required data in progress notes increased from 61 to 81% after an educational intervention [45].

#### User interface and workflow integration

Improving user interfaces is an important concern [10, 11, 39, 40, 42, 43]. The following issues should be addressed: unintuitive interfaces, complex navigation within the interfaces, insufficient user feedback, complex structured data capture.

Several user interface evaluation techniques were identified in the literature (Table 6).

**Table 6:** User interface evaluation techniques

Methods	Description
User testing [35, 39, 42]	EDR user testing with think-aloud process on pre-defined cases; recording of participants’ think-aloud statements and usability problems; data collection complemented with video, audio, or computer screen capture
Semiotic inspection method [40]	Deconstruction of EDR user interface into 3 sub-elements for analysis: metalinguistic signs, static signs, and dynamic signs
Cognitive task analysis [43]	Analysis of users’ cognitive activities when performing certain tasks like searching for information in an EDR
Real world observations [39]	Observational analyses in real clinical environment to provide insight on EDRs, clinical workflow, information gathering, and diagnostic decision making
Semi-structured interviews [39, 42]	Interviews aimed at assessing users’ experiences with EDRs, and in particular with SCCS, workflow, and interface
Survey method [42]	Questionnaires sent to users regarding improvements to be made on EDR usability domains

EDR: Electronic Dental Record; SCCS: Standardized Clinical Coding System

In their 2014 article, Walji et al*.* [42] evaluated 3 different methods (user testing, semi-structured interviews, and surveys) for detecting usability problems in an EDR. They concluded that the user testing method is better at detecting usability problems than the other two methods but that a combination of different complementary techniques is needed to provide a more comprehensive picture of EDR usability challenges.

According to [39, 42], issues related to EDR usability can be divided into three categories: user interface-related themes, SCCS-related themes, and work domain- and workflow-related themes.

More generally, the problems identified in the selected articles were: the lack of intuitiveness of the EDR interface, inadequate user guidance, and poorly organized controls. All of these were shown to impair users’ ability to determine how to perform a desired action and on which object the action should be performed [35, 40].

Lastly, the lack of easy and consistent access to patient data in most EDRs, and in particular the absence of an integrated view of the patient, requires users to switch between separate screens to see radiographs, intraoral photos, and clinical notes. This makes navigation cumbersome and introduces breakdowns in workflow [11, 38, 43, 44].

Other reported issues are summarized in Table 7.

**Table 7:** Issues related to the user interface and the workflow integration

Themes	Issues
User interface-related themes	Inadequate user guidance and poorly organized controls [35, 40] Ineffective feedback [42] Truncated label and text description in the EDR interface [39, 40, 42] Limited flexibility of user interface [35, 42, 44]
SCCS-related themes	Illogical ordering or classification of terms [39, 42] Non-searchable synonyms or alternative names for the same concept [39, 42] Confusion caused by abbreviations [39, 42]
Work domain- and workflow-related themes	Separation of clinically related information [35, 43, 44] Insufficient match between the user’s and the software application’s task model [35, 42]

EDR: Electronic Dental Record; SCCS: Standardized Clinical Coding System

#### Data capture solutions

Several studies proposed technical solutions to overcome difficulties in capturing data in EDRs.

In 2002, Chadwick et al*.* [34] suggested using barcodes to record undergraduate clinical activity in an academic environment. In 2009, Irwin et al*.* [36] developed and evaluated natural language processing aimed at extracting structured information from clinical notes to automatically annotate clinical notes with unified medical language system codes [46].

Finally, a voice-supported EDR was developed and tested in the field of temporomandibular joint disorders [37].

### Health information systems in dentistry and reuse of routine patient care data

Table 8 summarizes the articles selected in the last search.

**Table 8:** Articles selected in the search “health information systems in dentistry and reuse of routine patient care data”

References	Data reuse-related themes	Purpose(s)
Umar 2002 [59]	General, CDSS	General considerations on the reuse of captured data in the management of patient care
Mendonça 2004 [47]	CDSS	Discussion of the characteristics of CDSSs, the challenges in developing them, the potential barriers to their use in clinical practice, and the future perspectives opened by them
Khanna 2010 [48]	CDSS	Discussion of the technical challenges and future prospects associated with EDRs
Fricton et al*.*, 2011 [49]	CDSS	Evaluation of 2 CDSS activation approaches: the first based on an EDR and the second on the delivery of a secure e-mail or letter to patients encouraging them to ask their dental care provider to review the care guidelines specific to their medical condition
Rindal et al*.*, 2013 [60]	CDSS	Evaluation of computer-assisted guidance for dental office tobacco cessation counseling
Song et al*.*, 2013 [57]	Clinical search	Systematic review of studies on EDR data reuse in dental clinical research
Walji et al*.*, 2014 [58]	Clinical search	Introduction of BigMouth, a multi-institutional dental data warehouse
Chen et al*.*, 2016 [50]	CDSS	Introduction of an ontology-driven, case-based clinical decision support model for the design of removable partial dentures
Hunt et al*.*, 2017 [53]	Quality measure	Overview of the development of quality measures and discussion of their importance for improving clinical practice, notably in the academic context
Righolt et al*.*, 2019 [54]	Quality measure	Systematic review of studies describing existing quality measures in the field of oral health care or evaluating the scientific robustness and applicability of these measures
Obadan-Udoh et al*.*, 2019 [55]	Quality measure	Description of the unintended consequences and challenges of quality measurements in dentistry
Byrne et al*.*, 2019 [56]	Quality measure	Identification of the measures used to assess the quality of primary dental care and categorization of these measures according to the quality dimension to which they apply
Sayed 2019 [51]	CDSS	Systematic review of studies on CDSSs developed to help improve the survival of natural teeth
Machoy et al*.*, 2020 [52]	CDSS	Overview of the latest attempts to apply Artificial Intelligence (e.g., CDSSs or genetic algorithms) in research and clinical dentistry

CDSS: Clinical Decision Support System

Our scoping review found that routine patient care data are reused for three main purposes:

- In CDSSs, with a focus on patient care [47–52].
- In institutional or population-based health monitoring support systems, with a focus on the qualitative evaluation of care via quality measures [53–56].
- In clinical research, with a focus on the discovery of new knowledge [57, 58].

#### Clinical decision support systems

Clinical decision support systems are computer programs designed to provide expert support for health professionals making clinical decisions [47]. These applications may be standalone systems, or they may interact with and reuse data from other tools, including EDRs [47–49, 59].

In both our review and that of Sayed et al. [51], only three articles were found that proposed and evaluated a CDSS reusing EDR data [49, 50, 60] (Table 9).

**Table 9:** Clinical decision support systems reusing electronic dental record data

References	Type of EDR data	Conclusion
Fricton et al*.*, 2011 [49]	EDR data on patient conditions used for the development of specific care guidelines	The EDR-based CDSS increased the rate at which providers reviewed care guidelines and identified patients’ medical conditions
Rindal et al*.*, 2013 [60]	EDR data on patients who reported smoking cigarettes for providing patient centered evidence-based information	The EDR-based CDSS increased the rate at which providers assessed interest and discussed specific strategies for quitting and referred the patient to a tobacco quitline
Chen et al*.*, 2016 [50]	Structured EDR data on oral conditions of partially edentulous patients used for the design of removable partial dentures	The EDR-based CDSS facilitated the design of reasonable removable partial dentures based on similarity between instances in an ontology and patient cases

EDR: Electronic Dental Record; CDSS: Clinical Decision Support System

Studies of CDSSs concerns all areas of dentistry [52]. They rely on different data analysis techniques: namely, algorithmic systems, neural networks, probabilistic systems, logical/deductive systems, critiquing systems, model hybrid systems [52].

The main limitations of CDSSs are:

- The validity of CDSSs is mostly established internally, in narrow domains, and under varying conditions and technologies. Most CDSSs were not formally evaluated, and their value for clinical practice could not be established [47, 52].
- CDSSs are proliferating as fragmented and isolated systems with a few clinic- or hospital-wide exceptions in academic centers [47, 48].
- Structured data capture remains a challenge for all clinical information systems, including for CDSSs [47].

#### Quality measures

The National Quality Forum defines quality measures as “tools used to quantify the care provided to patients and gauge how improvement activities are indeed improving care or outcomes for certain conditions, in various settings, or during a specific timeframe” [53]. Quality measures concern all areas of health care delivery and population health, as defined by the National Quality Measures Clearinghouse: access, process, outcome, structure, use of service, health state, cost, and efficiency [53]. In 2011, the Health and Medicine Division [division of the National Academies of Sciences, Engineering, and Medicine] noted that the lack of quality measures acted as a barrier to improving oral health and reducing oral health disparities, and that quality measures in dentistry “lag far behind” those in medicine and other health professions [53–55].

Researchers, US state dental programs, and the US Dental Quality Alliance have sought to develop quality measures in the field of dentistry [53, 55, 56]. Except for some e-measures, all proposed measures were derived from administrative or claims-based data [55]. In the systematic review by Righolt et al*.* [54], only 2 out of 24 studies reused EDR data, specifically assessing the feasibility of an automated EDR-based quality measure (Table 10).

**Table 10:** Quality measures based on electronic dental record data

References	Quality measure	Measure specification organism
Bhardwaj et al*.*, 2016 [61]	Percentage of children who received fluoride varnish (CMS74v3)	Medicare and Medicaid services
Neumann et al*.*, 2017 [62]	Percentage of enrolled diabetic adults who received oral/periodontal evaluation within the reporting year (DOE-A-A)	Dental Quality Alliance

At present, the reuse of EDR data for quality measure development is far more common in the US than in Europe [53]. As EDR data are more detailed than claims data, they are considered more suitable for conducting quality measures [53]. Furthermore, the reuse of EDR data can advance quality measures through the automation of data collection, but also to increase transparency by availing access to information that would not be accessible otherwise [54]. However, the slow development of SCCSs and the increasing use of treatment procedure codes as a substitute for diagnosis severely limit both the reuse of EDR data for quality measure development and the ability to fully assess the impact of provided care [53].

It should be noted that Obadan-Udoh et al. [55] highlighted the ethical challenges posed by quality measures, in particular the risk of losing focus on the patient and that of compromising provider and patient autonomy.

#### Clinical research

**Fig. 5:**
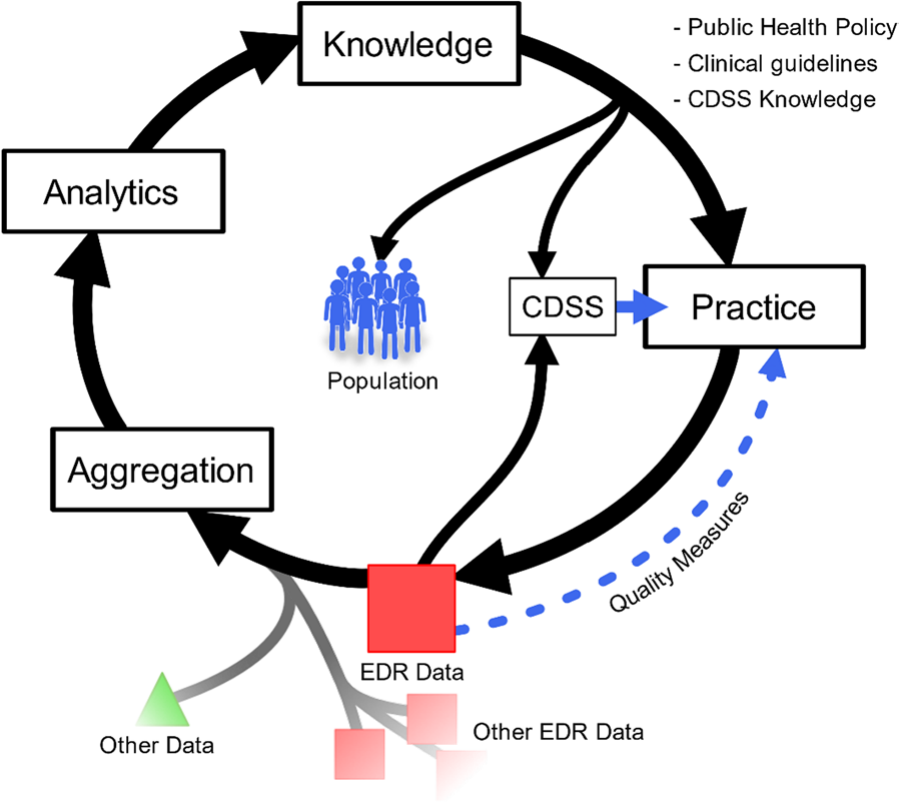
Learning Health Care System

The production of evidence-based knowledge using EDR data places the latter into a continuous cycle of improvement known as the Learning Health Care System [57]. Thus, after extraction, validation, and analysis, data from clinical practice can generate new knowledge, which in turn can influence clinical practice (Fig. 5).

In their systematic review, Song et al*.* [57] examined 60 studies that reused electronic patient data for dental clinical research. More than half of these studies addressed epidemiological topics, with a particular focus on the association between risk factors and various dental or medical conditions. All but two studies were retrospective, and most studies (72%) were conducted in the US.

The most frequently reported advantage of reusing EDR data is that they allow to conduct studies more effectively, at a lower cost, and with greater statistical power due to large sample sizes [57, 58]. Moreover, EDR data are deemed valuable because they constitute a rich resource for outcomes research. They help detect rare events or diseases and reduce study time [57, 58].

Over half of the studies examined in the systematic review by Song et al*.* [57] considered data availability and quality to be major limitations, as attested by the frequent presence of inaccurate, inconsistent, incomplete, or missing data. These limitations stem mainly from the fact that some data are not routinely documented in EDRs. However, they can also be attributed to coding errors or to inconsistent data documentation practices caused by the multiplicity of uncalibrated providers tasked with entering data [57].

Greater standardization of EDR data and increased adoption of public health databases and registries are needed to make EDR data more accessible in dental research [57]. In the US, a shared data warehouse named BigMouth was launched in August 2012, making data on 1.1 million patients available to users in the four contributing dental schools [58]. Nowadays, ten institutions have contributed to BigMouth by providing data on more than 3 million patients [63].

## Discussion

This scoping review traced all developments of the last decades in dental informatics, with a particular focus on SCCSs, data capture, and reuse of routine patient care data. To our knowledge, this is the first review to provide such a broad overview of the field.

### Principal findings

Most selected studies were conducted in the US. Righolt et al*.* and Song et al*.* [54, 57] made the same observation in their review of the literature on EDR data reuse. This finding points to a great disparity in the development of dental informatics, even among so-called developed countries.

The use of standardized codes and terms for treatment procedures is ubiquitous in many countries—e.g. Current Dental Terminology in the US, Uniform System of Coding and List of Services in Canada, *Classification Commune des Actes Médicaux* (Common Classification of Medical Acts) in France, and UPT codes in the Netherlands [25]. These codes are routinely used in dentistry to facilitate the recording of medical procedures in patients’ charts, the preparation of patient billing, and the transmission of data to third-party payers for patient reimbursement [18, 23]. Although these coding systems are comprehensive, they stay focused on treatment and do not allow to describe patients’ conditions. In view of this, another type of SCCS has been proposed in dentistry: DxTMs. Unlike free-text notes, DxTMs allow HIS users to directly access information on patients’ conditions, track clinical outcomes, monitor best practices, and develop evidence-based guidelines [23, 27].

In the field of medicine, DxTMs have been in use for decades [18]. The best known DxTM is the ICD, which was adopted in 1900 as an international standard for describing diagnoses (it was then called the International List of Causes of Death) [32]. From the start, oral health diagnoses were hardly represented in this ICD. Efforts were initially made to include new diagnoses, and in 1969 the first version of the Application of the ICD to Dentistry and Stomatology (ICD-DA) was issued [64]. Nevertheless, some authors have highlighted the inadequacy of the existing ICD-DA terminology for oral diagnosis documentation [17, 32].

**Fig. 6:**
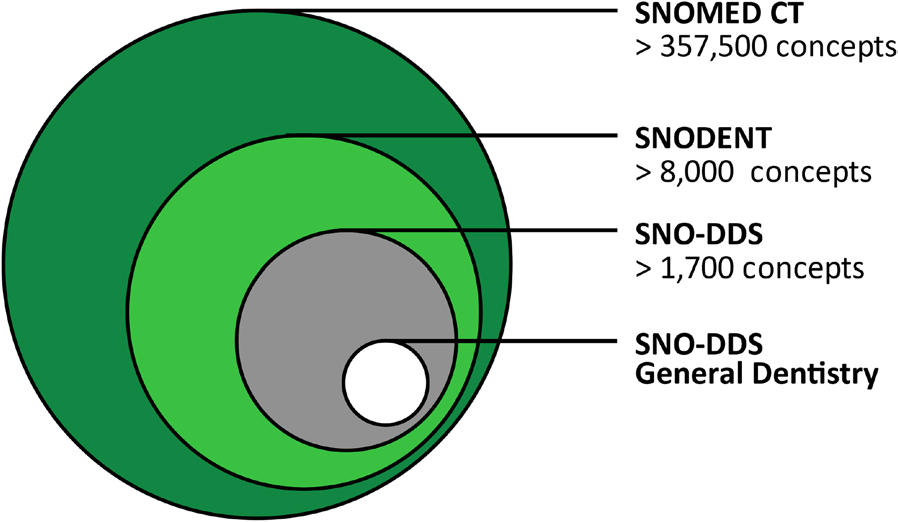
SNOMED CT and its subsets [14, 32, 65]

In addition to ICD revisions, several proposals have been made to adapt SCCSs to dentistry. Of these, two ontologies are now available for use: SNOMED CT (and its subsets) (Fig. 6) and the Oral Health and Disease Ontology.

Recently, some of the Oral Health and Disease Ontology developers have begun to participate in the revision and development of SNODENT. Therefore, a rapprochement between SNODENT and the Oral Health and Disease Ontology is likely, especially in terms of their use and implementation [66].

In the academic setting, the use of SCCSs can help reinforce student reasoning of why specific procedures need to be performed. Some EDRs used in dental schools allow students to enter three types of diagnoses: tentative, working, and definitive [18]. This process is of prime pedagogical interest as it allows to appreciate students’ clinical reasoning and its evolution over time. Likewise, determining the plausibility of the entered diagnostic code based on the entered treatment procedure code opens interesting pedagogical perspectives. More generally, SCCSs can be used to monitor teaching and knowledge acquisition at both the collective and individual levels. They can also highlight gaps in teaching or ambiguities in diagnostic classification, as shown by Sutton et al. [28], who highlighted the need for faculty member calibration in the area of dental caries classification diagnosis. Reed et al. [26] found that the use of SCCSs has a positive impact on the education of dental students. This is consistent with the findings of a study on the pedagogical value of using SCCSs in other medical fields [67].

Although SCCSs have been developed specifically to describe patients’ conditions, their validation in practice is essential. Indeed, EDR data must be consistent with clinical reality to limit the need for post-capture data cleaning and to ensure correct inferences [25]. In order to fulfill their role effectively, EDRs require complete and accurate capture of clinical data [10], with a view to their possible reuse outside of care.

The semantic and syntactic proximity of certain terms tends to result in misuse, especially when users are not trained. These terms should therefore come with extra textual definitions. This is not the case yet for SNODENT, and the question of what SNOMED terms actually represent remains a matter of debate [66].

Moreover, the complexity of SCCSs makes them difficult to navigate from the EDR user interface, which can negatively impact data capture.

Many issues can thwart data capture: a lack of universally accepted documentation standards and information needs, incomplete or inaccurate record practices, lack of usability of EDR user interfaces, a lack of easy and consistent access to patient data, and inaccurate workflow integration [10, 12, 39, 44]. These various problems, both technical and socio-organizational, condition the usability of EDRs [11, 35, 39]. One of the main challenges of data capture is to ensure the customization of EDRs according to dental department needs or user expectations [11, 43].

Practitioners typically spend more time than they would like in properly documenting clinical information, which reduces the time available for patient care or other activities [10]. The need to improve user interface has been identified as a major concern in the literature, as has the need to streamline workflow integration [10, 11, 39, 40, 42, 43]. For this purpose, EDRs should support the entire process of care by enabling the capture and display of all necessary information at the right time. Health care teams could adapt to simplify data capture and free up practitioners’ time. For example, dental assistants or patients could fill out forms electronically before appointments, such that practitioners would only need to review, update, and pull the information into the EDR [10]. However, overly important adaptation requirements, missing functionalities, and incompatibilities with clinical practice can generate workflow problems [39]. This requires taking into consideration practitioners’ environment, habits, and clinical specialization. Although it is difficult to customize EDRs to each practitioner, there is a need for EDRs that can adapt to the most common situations.

No study on the specifics of dental practitioners’ working environment was identified in our review. This is unfortunate, as the dental chair would benefit from being interfaced with the EDR. This would greatly facilitate the integration of data capture into processes at multiple levels (care, radio examination, etc.).

As regards care data reuse, EDRs have received increased attention because they help to expand evidence-based knowledge [57], assess needs, and improve quality of care. The most obvious obstacles to the widespread reuse of EDR data are the lack of standards in dentistry and data availability and quality issues [55, 57, 58]. The reuse of EDR data can complement traditional research methods or can function in synergy with them [57]. In the US, the work carried out on SCCSs has allowed for significant progress, in particular with the creation of the shared data warehouse BigMouth [58].

In addition, EDR data can be used to evaluate health changes and to monitor dental service utilization, care delivery, and disparities between treatment needs and provision [55, 57]. While the data most frequently used for this purpose are derived from dental insurance claims, EDRs allow for better quality measures because they contain more detailed data on patients [53]. The World Dental Federation has defined quality as an “iterative process involving dental professionals, patients, and other stakeholders to develop and maintain goals and measures to achieve optimal health outcomes” [56]. This definition highlights the need to develop quality measures in dentistry to improve patient care, but also to reduce costs and enhance patient experience [53–56]. Despite the interest in using EDRs for quality measure development, particularly in the areas of data collection automation and transparency, only two publications were found that examined quality measures based on EDRs [54]. According to Hunt et al*.* [53], faculty should implement quality measurement processes in their clinical programs to prepare graduates for their future practice.

Lastly, EDRs can be used with CDSSs to improve the quality of care. Indeed, CDSSs can provide real-time quality assurance, support treatment planning, generate alerts, or remind clinicians of the need to perform routine tasks for patients with potentially risky conditions [47, 48]. Despite the recognized need for CDSSs, their implementation has been limited by a lack of formal evaluation, challenges in developing standard representations, a lack of studies on decision-making processes, the cost and difficulties involved in the generation of a knowledge base, practitioner skepticism about the value and feasibility of decision support systems, etc. [48]. Ideally, CDSSs should be integrated into EDRs, as this allows physicians to capture data only once, without extra costs or workflow breakdown. Within the limits of this scoping review, only three studies proposed an EDR with an integrated CDSS [49, 50, 60].

## Limitations

The main limitation of this review lies in the search strategy, which may have prevented us from identifying all studies of interest due to limitations in database coverage and to the particularities of article indexing. To compensate this limitation a manual bottom-up search of the references of each selected article was performed.

Regarding the reuse of routine patient care data, our work aimed only to include studies addressing data reuse in view of information system. Therefore, studies that reuse electronic data without consideration of HIS were excluded from this scoping review.

Another area of research related to data reuse is the Dental Practice-Based Research Network (DPBRN). The DPBRN brings together solo and large group practitioners to accelerate the development and conduct of clinical studies on important issues in oral health care [68]. In this scoping review, no articles concerning the DPBRN could be included due to the bibliographic search strategy or the inclusion and exclusion criteria. Although in 2013, the overwhelming majority of dental PBRN studies used paper forms for data [68], some studies determined the feasibility of conducting clinical research using the electronic dental record within DPBRN [69, 70].

## Conclusion

Since its introduction in 1968, dental informatics has gradually developed in the field of HISs and has tried to catch up with advances in medicine. As was the case in the medical setting, the various issues raised by the standardization, capture, and reuse of data had to be addressed. In addition to technical difficulties, HISs present socio-organizational problems linked to workflow integration, human–machine interface, the use of EDRs in the academic setting, and scientific and ethical issues.

Given the new paradigm of clinical data reuse outside of care, it is necessary to maximize both ease-of-use and the workflow integration of EDR data capture [10]. It also seems important to give practitioners access to data or indicators such as quality measures to allowing them to manage and improve their clinical activity, but also encourage them to capture data in an optimal way.

Efforts in terms of standardization and interoperability have led to concrete progress that allows EDR data to be aggregated with other health and non-health data (e.g., geographic data) to generate new broader knowledge. Despite these advances, strong governance seems fundamental to achieve concrete achievements such as inter-university data warehouses [71].

Some studies selected in this review assessed the educational value of HISs, but only in relation to SCCSs. The latter can indeed be used to evaluate students’ diagnostic abilities in a clinical situation. Clinical data linked to care provided by students would benefit from being exploited.

They could be used to monitor the acquisition of student skills at both the technical and intellectual levels or to ensure that students properly perform enough procedures to be able to have their own private practice. Moreover, HISs in dentistry can solve problems that are specific to academic research centers. For instance, they can reduce waiting times linked to teachers’ successive validations of the different stages of treatment performed by students [72].

In conclusion, there is a need for greater development of dental informatics in the field of HISs and for further studies on their educational value.

## Supplementary Information

**Additional file 1**. Minimum Clinical Documentation Checklist by Tokede *et al.*

## Data Availability

Not applicable.

## Abbreviations

HIS: Health information system
EDR: Electronic dental record
SCCS: Standardized clinical coding system
ICD: International Classification of Diseases
CDSS: Clinical decision support system
PRISMA-ScR: Preferred Reporting Items for Systematic Reviews and Meta-Analyses extension for Scoping Reviews
OBO: Open biomedical ontologies
DDS: Dental diagnostic system
HSRT: Health sciences reasoning test
DPBRN: Dental practice-based research network
DxTM: Standardized diagnostic terminology
