# Correction to: Current state of dental informatics in the field of health information systems: a scoping review

**DOI:** 10.1186/s12903-022-02221-2

**Published:** 2022-05-28

**Authors:** Benoit Ballester, Frédéric Bukiet, Jean-Charles Dufour

**Affiliations:** 1 Pôle d’Odontologie, Assistance Publique des Hôpitaux de Marseille, Marseille, France; 2 Aix Marseille Univ, CNRS, ISM, Inst Movement Sci, Marseille, France; 3 Aix Marseille Univ, INSERM, IRD, SESSTIM, Sciences Economiques & Sociales de la Santé & Traitement de l’Information Médicale, ISSPAM, Marseille, France; 4 APHM, Hôpital de la Timone, Service Biostatistique et Technologies de l’Information et de la Communication, Marseille, France

## Correction to: Benoit et al. BMC Oral Health (2022) 22:131 10.1186/s12903-022-02163-9

The given name and family name of all the authors were reversed in the original version of the publication. It has been corrected with this erratum.

