# Supplementary material for: Current state of dental informatics in the field of health information systems: a scoping review
Source: BMC Oral Health. 2022 Apr 19;22:131. doi: 10.1186/s12903-022-02163-9 (PMC9020044; doi:10.1186/s12903-022-02163-9)
Supplement: Supplementary file 1 — Additional file 1. Minimum Clinical Documentation Checklist by Tokede et al. [file 12903_2022_2163_MOESM1_ESM.docx]

## Appendices

### Appendix A

Summary of responses from the second round of the Delphi process on what data should be entered in electronic dental records and on how often each clinical entry should be updated [10]

| **Minimum Clinical Documentation Checklist** | **Agreed Frequency** | **Round 2**  **Agreement (%)** |
| --- | --- | --- |
| Update Medical History | Every Visit | 100 |
| Update Dental History | Every Visit | 89 |
| Perform Intra-oral examination and Oral Cancer Screening | Bi-annually | 84 |
| Perform extra-oral examination | Bi-annually | 79 |
| Record Chief Complaint | Every Visit | 89 |
| Update Medication and Allergy History | Every Visit | 89 |
| Review radiographs | When Needed | 100 |
| Record vital signs | Every Visit | 84 |
| Obtain informed consent | When Needed | 100 |
| Review treatment plan | Every Visit | 95 |
| Record dental diagnosis | Every Visit | 89 |
| Record treatment/clinic notes | Every Visit | 100 |
| Update odontogram (tooth chart) | Bi-annually | 100 |
| Update periodontal chart | Bi-annually | 100 |
